# Prognostic and predictive biomarkers for response to neoadjuvant chemoradiation in esophageal adenocarcinoma

**DOI:** 10.1186/s40364-022-00429-6

**Published:** 2022-11-14

**Authors:** Hirsch Matani, Divya Sahu, Michael Paskewicz, Anastasia Gorbunova, Ashten N. Omstead, Rodney Wegner, Gene G. Finley, Blair A. Jobe, Ronan J. Kelly, Ali H. Zaidi, Ajay Goel

**Affiliations:** 1 Division of Radiation Oncology, Allegheny Health Network Cancer Institute, Pittsburgh, PA USA; 2 Department of Molecular Diagnostics and Experimental Therapeutics, Beckman Research Institute of City of Hope, 1218 S. Fifth Avenue, Suite 2226, Biomedical Research Center, Monrovia, CA 91016 USA; 3 Esophageal and Lung Research, Allegheny Health Network, Pittsburgh, PA USA; 4 Department of Hematology and Oncology, Charles A. Sammons Cancer Center, Baylor University Medical Center, Dallas, TX USA

**Keywords:** Esophageal adenocarcinoma, Risk stratification, CROSS regimen, EPHA5, BCL6, ERBB2, Predict response, Prognostic classifier

## Abstract

**Background:**

Esophageal adenocarcinoma is a lethal disease. For locally advanced patients, neoadjuvant chemoradiotherapy followed by surgery is the standard of care. Risk stratification relies heavily on clinicopathologic features, particularly pathologic response, which is inadequate, therefore establishing the need for new and reliable biomarkers for risk stratification.

**Methods:**

Thirty four patients with locally advanced esophageal adenocarcinoma were analyzed, of which 21 received a CROSS regimen with carboplatin, paclitaxel, and radiation. Capture-based targeted sequencing was performed on the paired baseline and post-treatment samples. Differentially mutated gene analysis between responders and non-responders of treatment was performed to determine predictors of response. A univariate Cox proportional hazard regression was used to examine associations between gene mutation status and overall survival.

**Results:**

A 3-gene signature, based on mutations in EPHA5, BCL6, and ERBB2, was identified that robustly predicts response to the CROSS regimen. For this model, sensitivity was 84.6% and specificity was 100%. Independently, a 9 gene signature was created using APC, MAP3K6, ETS1, CSF3R, PDGFRB, GATA2, ARID1A, PML, and FGF6, which significantly stratifies patients into risk categories, prognosticating for improved relapse-free (*p* = 4.73E-03) and overall survival (*p* = 3.325E-06). The sensitivity for this model was 73.33% and the specificity was 94.74%.

**Conclusion:**

We have identified a 3-gene signature (EPHA5, BCL6, and ERBB2) that is predictive of response to neoadjuvant chemoradiotherapy and a separate prognostic 9-gene classifier that predicts survival outcomes. These panels provide significant potential for personalized management of locally advanced esophageal cancer.

**Supplementary Information:**

The online version contains supplementary material available at 10.1186/s40364-022-00429-6.

## Background

Esophageal cancer (EC) is the 7th most commonly diagnosed cancer (3.2% total new cases per annum) and the 6th leading cause of cancer-related deaths (5.3% total death per annum) worldwide [1]. In the western hemisphere, the most common histological subtype is esophageal adenocarcinoma (EAC). Despite major advances in surgical approaches to locally advanced EC, the 5-year survival of patients treated with curative resection remains poor due to distant and locoregional recurrence of the disease [2, 3]. Consequently, neoadjuvant therapy, either trimodality approaches involving chemoradiotherapy (CRT) followed by esophagectomy or perioperative chemotherapy before and after surgery, has become a standard with the intent to eradicate occult micrometastatic disease and improving both survival and surgical outcomes. In the United States, the preferred approach for locally resectable disease based on the NCCN Guidelines is CRT (CROSS regimen consisting of paclitaxel, carboplatin, and 41.4 Gy / 23 fractions) followed by surgery [4].

The CROSS regimen is based on the data from a randomized control trial of 368 patients, of which 75% were adenocarcinoma subtype. Overall, the study demonstrated for the neoadjuvant group a pathologic complete response (pCR) rate of 29%, complete resection (R0) rate of 92%, and a median overall survival of 48.6 months compared to 24 months for surgery alone (HR 0.68 [95% CI 0.53–0.88]; *p* = 0.003). Additionally, the CRT arm demonstrated no significant difference in perioperative complications or surgical mortality compared to surgery alone. Therefore, the afforded survival benefit has been attributed solely to CRT [5]. Unfortunately, not all esophageal cancer patients treated with this multimodality approach will benefit, resulting in a sizable proportion of patients demonstrating limited clinical benefit. In this context, it is beneficial to identify new and improved predictive/prognostic classifiers for accurate stratification and individualization of multimodality treatment for patients with locally advanced esophageal cancer [6].

Current risk stratification is primarily based upon clinicopathologic features, including TNM staging, which is inadequate [7]. With pCR being the strongest objective classifier; a small subset of patients with pCR have a five-year survival rate advantage of approximately 60%, making it a known proxy for favorable outcomes [8, 9]. In addition, adverse resected tumor pathology, including poor differentiation, mucinous or signet ring histology, and extensive lymphovascular involvement are associated with adverse clinical outcomes [10, 11].

In this study, we aim to identify novel and ubiquitous genetic classifiers that either predict response to neoadjuvant CRT or predict survival outcomes in patients afflicted with locally advanced EAC. Overall, the development of better predictive/prognostic classifiers could help patients avoid unnecessary toxicity from neoadjuvant therapy, streamline curative therapy, and limit delays in surgery. Additionally, identification of pCR status upfront may lead to esophageal preservation and better quality of life in select responders. Similarly, the development of better prognostic classifiers can improve outcomes by helping to stratify poor responders to adjuvant therapy.

## Methods

### Study population

This study was approved by the Allegheny General Hospital IRB and no written informed consent was obtained from participants as the study was a retrospective review. Genetic sequencing was performed on paired pre- and post-tissue samples acquired from 34 patients undergoing neoadjuvant therapy for locally advanced EAC. The diagnostic FFPE blocks were obtained from pathology to power the analysis. Tumors were assigned a pathological tumor, node, and metastases stage as defined by the American Joint Committee on Cancer 7th edition. For the predictive analysis, we excluded thirteen patients who did not receive carboplatin/paclitaxel with radiation or for whom both pre-and post-treatment genetic analyses were not performed. Response to therapy was classified as partial or complete response. Non-response was classified as stable disease or progression.

### DNA extraction

A total of 55 paraffin-embedded tumor tissues were macrodissected, and genomic DNA was extracted using a QIAamp DNA FFPE Tissue Kit (Qiagen, Valencia, CA, United States). Tissue slides were baked at 68 °C for 20 to 30 s followed by 3 times treatments with xylene to deparaffinized, and residual xylene was removed by washing through serial dilutions of ethanol. Next, the tumor tissues were placed in tubes and allowed to dry into pellets that were resuspended in Buffer ATL with added proteinase K. The procedures following this were in accordance with Qiagen’s protocol guidelines. Each resulting DNA specimen was assessed by both Qubit and TapeStation analysis to determine both the quantity and quality of the template including average genomic fragment length and DNA Integrity Number (DIN) performance prediction. Normal cutoff thresholds for DNA quantity and integrity were used as previously described [12].

### Targeted Next Generation Sequencing (NGS)

Targeted NGS was performed as described previously [13]. SureSelect-XT Target Enrichment Kit, https://www.agilent.com/en/product/next-generation-sequencing/hybridization-based-next-generation-sequencing-ngs/dna-seq-reagents-kits-library-preparation-kits/sureselectxt-reagent-kits-232859 (Agilent Technologies, Santa Clara, CA, United States) was used to prepare the DNA libraries. Briefly, 250 ng DNA was fragmented to a size of 250 to 300 bp, by a Covaris M220 sonicator. The DNA fragments were end-repaired and A-tailed, followed by adaptor ligation and subsequent amplification of the ligated DNA fragments using 10 cycles of polymerase chain reaction (PCR). Each amplified library was then hybridized to a SureSelect 640 genes oncogenesis custom panel 2.8 Mb bait set (Agilent Technologies, Santa Clara, CA, United States) according to the manufacturer's protocol (Individual gene list provided as supplementary material). Captured DNA was washed and amplified by 12 PCR cycles following the manufacturer's guidelines. Next, Tapestation 2200 (Agilent Technologies, Santa Clara, CA, United States) was used to assess the size and concentration of the captured DNA. Captured samples were pooled and sequenced on a single HiSeq flow cell on HiSeq 2500 (Illumina, San Diego, CA, United States), using a 2 × 100 bp PE Rapid Run v2 protocol.

### Next generation sequencing hybrid capture analysis

FASTQ files were generated from Binary Cluster Files (.bcl) using manufacturer-provided demultiplexing software, bcl2fastq v1.8.4 with parameters recommended by the manufacturer. The resulting FASTQ files were then aligned to the human genome reference hg19 (GRCh37) using the Burrows-Wheeler Aligner v0.7.10 algorithm [14, 15] with default settings. PCR duplicate marking and read pair insert size estimation was performed using Picard Tools 39 (v1.125) [16]. Resulting in alignment files in bam and bai formats being used for further downstream processing. Variants were called using an in-house variant caller algorithm (Johns Hopkins University, Baltimore, MA, United States; # MDLVC v7.5) cross-referenced with HaplotypeCaller (Genome Analysis Tool Kit 3.3) [17] under discovery mode across coding and splice sites. Variants passing the 5% variant allele frequency filter and with a minimum of 50 × allele depth were retained for further analysis. Variants were annotated for genomic regions using annovar [18] (version 07042018) and with COSMIC [19] and dbSNP [20] to know possible somatic and germline status. Variant calls falling in non-coding regions were excluded from the analysis. Further variants that are designated with dbSNP common polymorphism status or failing laboratory quality control such as a pool of normal artifact threshold were excluded from the analysis. The resulting final variant calls were used for further downstream analysis.

### Generation of mutation annotation data

To convert a variant call format (VCF) file into a mutation annotation format (MAF) file we used a publicly available vcf2maf.pl Perl script https://github.com/mskcc/vcf2maf#vcfmaf. The MAF generated for pre-treatment and post-treatment samples containing somatic mutations were summarized, analyzed, and visualized using the *R* Bioconductor package Maftools [21]. We next performed differentially mutated gene analysis between responders and non-responders from the pre-treatment samples using the mafCompare function. A threshold of mutation in two samples in at least one of the cohorts was used subsequently for filtration, with a fisher’s exact *P* value < 0.05.

### Selection of nonsynonymous mutations and construction of mutation matrix

We considered nonsynonymous somatic mutations including frameshift deletion, frameshift insertion, splice site, translational start site, nonsense mutation, missense mutation, nonstop mutation, in-frame deletion, and in-frame insertion for the construction of the mutation matrix. The mutation data for these somatic mutations for each gene were extracted and used for mutation matrix construction. The mutation matrix is an *m* × *n* matrix where *m* indicates the number of patients from the pre-treatment samples and *n* represents the number of genes. Values in the mutation matrix specify the presence of any of these nonsynonymous mutations as 1 or the absence of these mutations as 0 in a gene in one patient, respectively.

### Detection of prognostic mutated genes

Univariate Cox proportional hazard regression was applied to examine the association between genes, nonsynonymous mutational status, and patient overall survival (OS). Mutated genes with statistically significant association (*P* < 0.05) with patient OS were selected and integrated into a mutational risk score formula. The risk score for each patient was calculated by a linear combination of univariate coefficient and mutational status of the gene as follows:

$$Mutational\;Risk\;Score=\sum_{j=1}^nW_j\ast{mut.status}_{ij}$$

*W*_*j*_ is the univariate coefficient for gene *j*, *mut.status*_*ij*_ is the presence or absence of nonsynonymous mutation of gene *j* in patient *i,* and *n* is the number of mutated genes. Here, *n* is 9.

### Statistical analysis

Kaplan–Meier survival analysis (e.g., low vs. high mutational risk score group) with Mantel log-rank test was performed for the difference between survival curves. Survival analysis was performed using the survival *R* package [22]. Receiver operating characteristic curve (ROC) curve and area under curve (AUC) analyses were performed to evaluate the sensitivity and specificity of the mutated genes risk score for OS predictions. The AUC, *P* value and confidence interval for AUC were calculated using the MedCalc software version 19.2.1. All statistical tests were two-sided.

## Results

### Patient characteristics

**Fig. 1:**
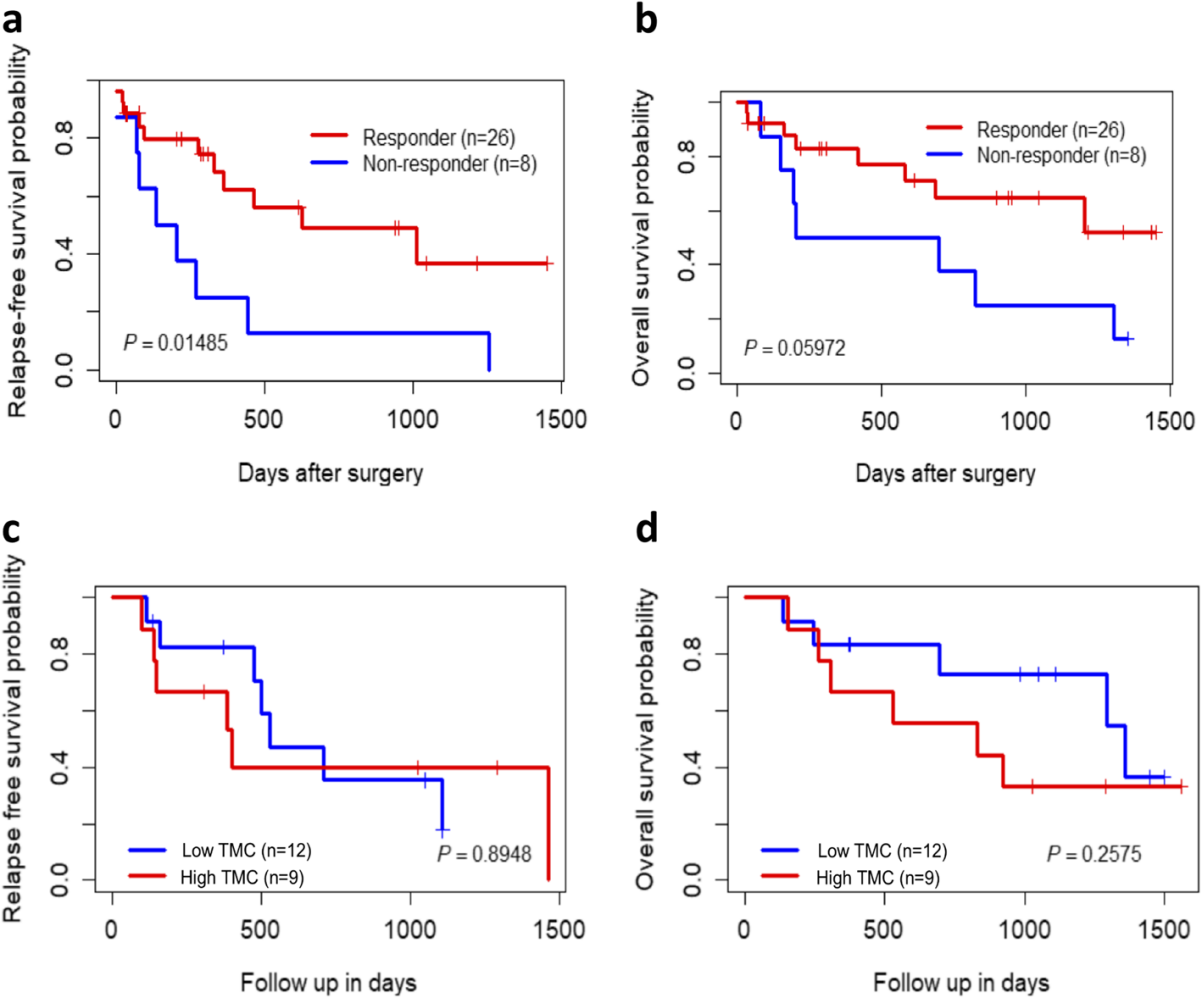
Survival estimates based on response to neoadjuvant chemotherapy and tumor mutation count in EAC. Kaplan–Meier plots of responder and non-responder group of patients for neoadjuvant chemotherapy (**A**) relapse-free survival (**B**) overall survival. Kaplan–Meier plots of low and high tumor mutation count based on median tumor mutation count of the cohort (**C**) relapse-free survival (**D**) overall survival. Here, TMC is tumor mutation count

Thirty-four patients were included in the prognostic analysis and 21 patients of these patients were included in the predictive analysis, treated from October 2013 to April 2018. The mean patient age was 64 years and the vast majority (97%) of patients were male. Primary tumor staging at diagnosis was as follows: T1 and T2 *n* = 7 patients (20.5%), T3 *n* = 25 patients (73.5%), and unknown *n* = 2 patients (6%). Lymph node staging at diagnosis was as follows: N0 *n* = 9 patients (26.5%), N1 *n* = 10 patients (29%), N2 *n* = 7 patients (20.5%), N3 *n* = 4 patients (12%), and unknown *n* = 4 patients (12%). The median radiation dose delivered was 45 Gy (4100–5040). In the overall cohort, 41% of patients had a complete response (*n* = 15) and 29% had a partial response (*n* = 11) to neoadjuvant therapy compared with 29% (*n* = 6) and 43% (*n* = 9), respectively for those patients receiving CRT with carbo/taxol. Additionally, for the complete dataset response to neoadjuvant treatment was an independent predictor of relapse-free survival (*p* = 1.49E-02) but not for overall survival (*p* = 5.97E-02) – Fig. 1A and B.

Finally, 57% of patients with node-positive disease at diagnosis had complete resolution of nodal disease at the time of surgery. Median overall survival was 45.3 months (95% CI 26 -52.1) **–** Table 1.

**Table 1:** Baseline patient characteristics (*n* = 34)

Age Mean (SD)	64 (8)
Characteristics	No. (%)
Gender	
Male	33 (97)
Female	1 (3)
T Stage at diagnosis	
T3	25 (73.5)
< T3	7 (20.5)
Unknown	2 (6)
N Stage at Diagnosis	
0	9 (26.5)
1	10 (29)
2	7 (20.5)
3	4 (12)
Unknown	4 (12)
Resection Margin (R0)	
Yes	31 (91)
No	3 (9)
Neoadjuvant Treatment	
Carboplatin, Paclitaxel and Radiation	21 (62)
Other	13 (38)
Radiation Dose	
≤ 4140 cGy	4 (12)
> 4140 cGy	22 (59)
Unknown	2 (6)
None	6 (24)
Pathologic Response	
Stable Disease	5 (15)
Partial Response	11 (29)
Progressive Disease	3 (9)
Complete Response	15 (41)
Recurrence	
Yes	19 (56)
No	15 (44)
Mortality	
Alive	18 (53)
Deceased	16 (47)
Type of Surgery	
Minimally Invasive Esophagectomy	29 (85)
Other	5 (15)
Age Mean (SD)	64 (8)

### Predictors of response to CROSS regimen

**Fig. 2:**
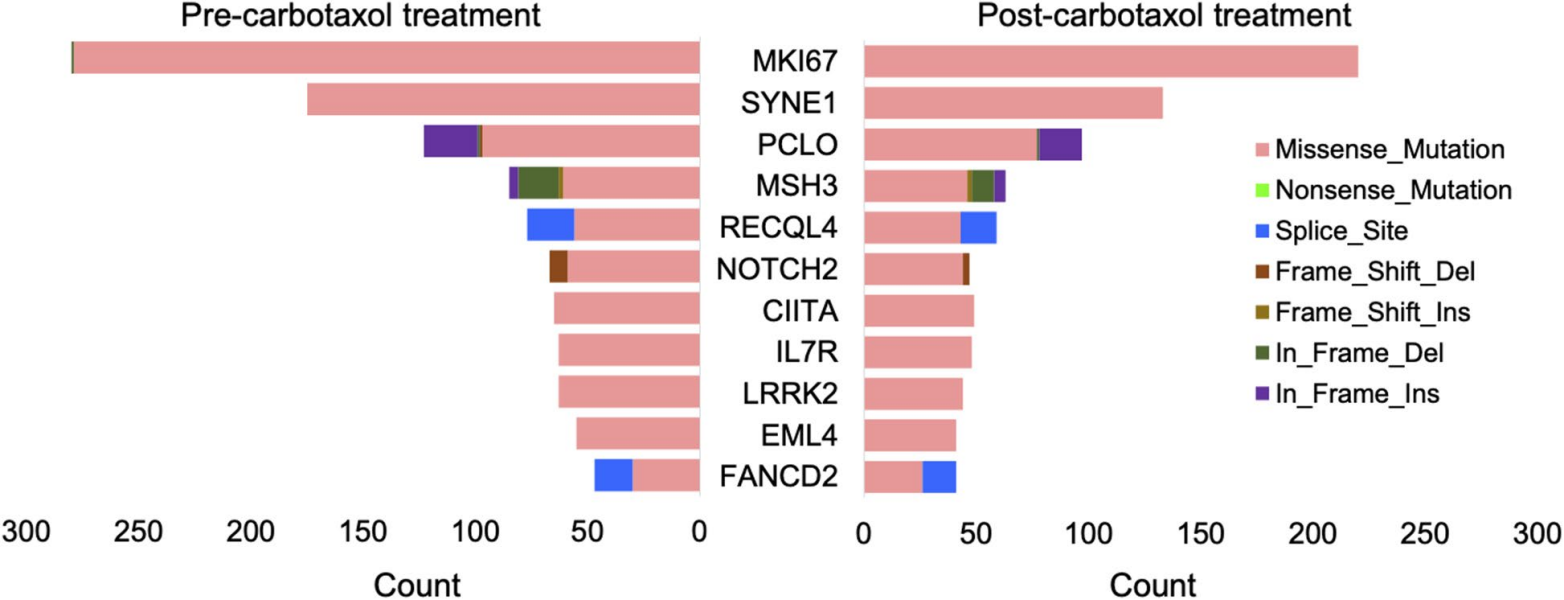
Frequency of non-synonymous mutations in the top 10 frequently mutated genes in pre- and post- treatment with carboplatin/paclitaxel and radiation in patients with EAC

Among the subset of responders to CRT only, the most frequently mutated genes were MKI67, SYNE1, PCLO, MSH3, RECQL4, NOTCH2, ILR7, CIITA, LRRK2, and EML4. Tumor mutation count was significantly reduced for these genes in post-treatment samples compared to pre-treatment samples (*p* = 5.89E-03). For responders, the top 10 mutated genes were common for pre- and post-treatment samples except NOTCH2 mutations found in pre-treatment samples and FANCD2 mutations found in post-treatment samples only. Non-responders harbored frequent mutations in NLRP1, ALK1, and MAP3K1. The total number of mutations was significantly reduced for these genes in post-treatment samples as well (*p* = 4.85E-03). The majority of these mutations across both responders and non-responders were missense mutations (single nucleotide polymorphism) and C > T was the most common single nucleotide variant – Fig. 2.

Tumor mutation count was not an independent predictor of relapse-free survival (Fig. 1C) or overall survival (Fig. 1D) in patients treated with the CROSS regimen.

**Fig. 3:**
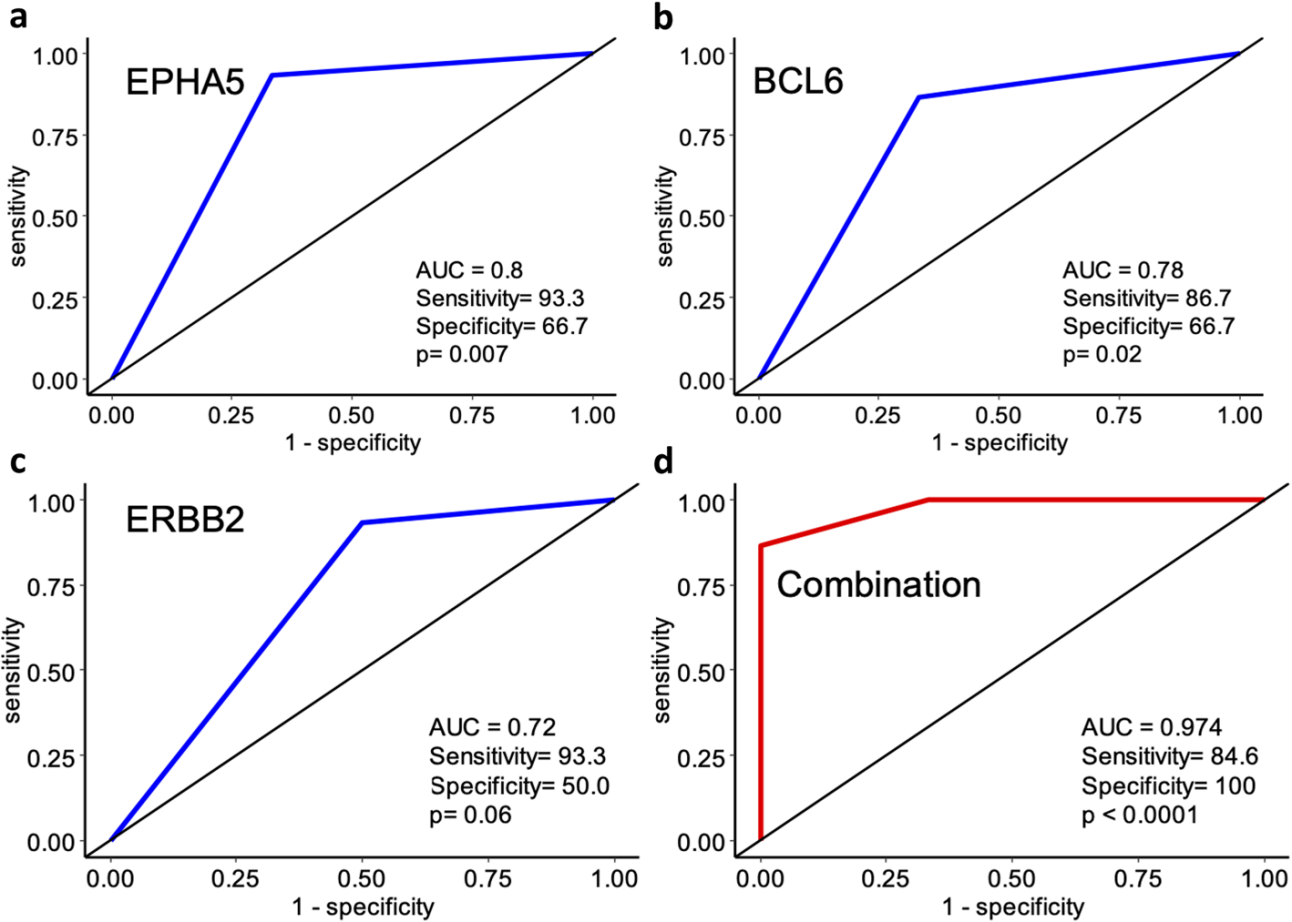
ROC curve analysis of response to carboplatin/ paclitaxel based chemoradiation prediction by the differentially mutated genes in EAC. ROC curve shows high sensitivity and specificity for predicting response to chemoradiotherapy. **A** EPHA5 gene (**B**) BCL6 gene (**C**) ERBB2 gene (**D**) Combined 3-gene signature

Overall, fifteen patients responded to the CROSS regimen while 6 patients had stable or progressive disease. Differentially mutated gene analysis utilizing pretreatment biopsies suggests that mutations in EPHA5 (*p* = 7.00E-03; Fig. 3A) and BCL6 (*p* = 2.00E-02; Fig. 3B) predict resistance to, whereas mutations in ERBB2 (*p* = 6.00E-02; Fig. 3C) predict response to CRT. Importantly, the combination of these three genes has significantly enhanced the prediction accuracy of CRT in treatment naïve biopsies (AUC = 0.974, *p* < 0.0001; Fig. 3D). For this model, sensitivity was 84.6% and specificity was 100%.

### Prognostic classifiers for locally advanced EAC

**Fig. 4:**
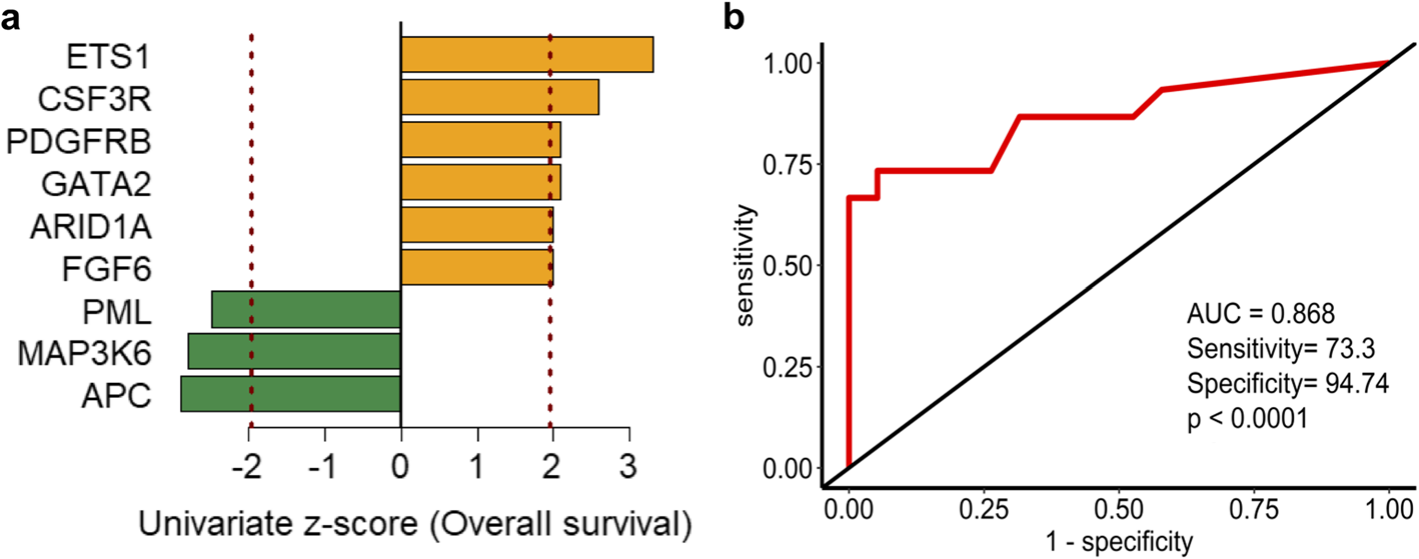
Univariate Cox regression of prognostic mutated genes (**A**). Bar graph shows 9 prognostic mutated genes ordered by their univariate z-score for overall survival. A positive score indicates nonsynonymous mutations in the genes are associated with shorter survival, and negative scores are associated with longer survival. The dashed line (colored in red) represents an absolute univariate z-score value of ± 1.96. ROC curve analysis of overall survival prediction by the 9-mutational gene signature in patients with EAC (**B**)

In the entire 34-patient cohort, 9 genes with univariate Cox analysis were statistically significantly (*p* < 0.05) associated with overall survival. The three genes in which the presence of nonsynonymous mutation with a negative coefficient was associated with improved patient survival APC (*p* = 4.73E-03), MAP3K6 (*p* = 1.90E-03), and PML (*p* = 7.00E-03). The remaining six genes with positive coefficient, in which nonsynonymous mutation was associated with worst patient survival such as ETS1 (*p* = 2.457E-05), CSF3R (*p* = 5.00E-03), PDGFRB (*p* = 2.10E-02), GATA2 (*p* = 3.00E-02), ARID1A (*p* = 3.00E-02) and FGF6 (*p* = 3.70E-02) – Fig. 4A.

**Fig. 5:**
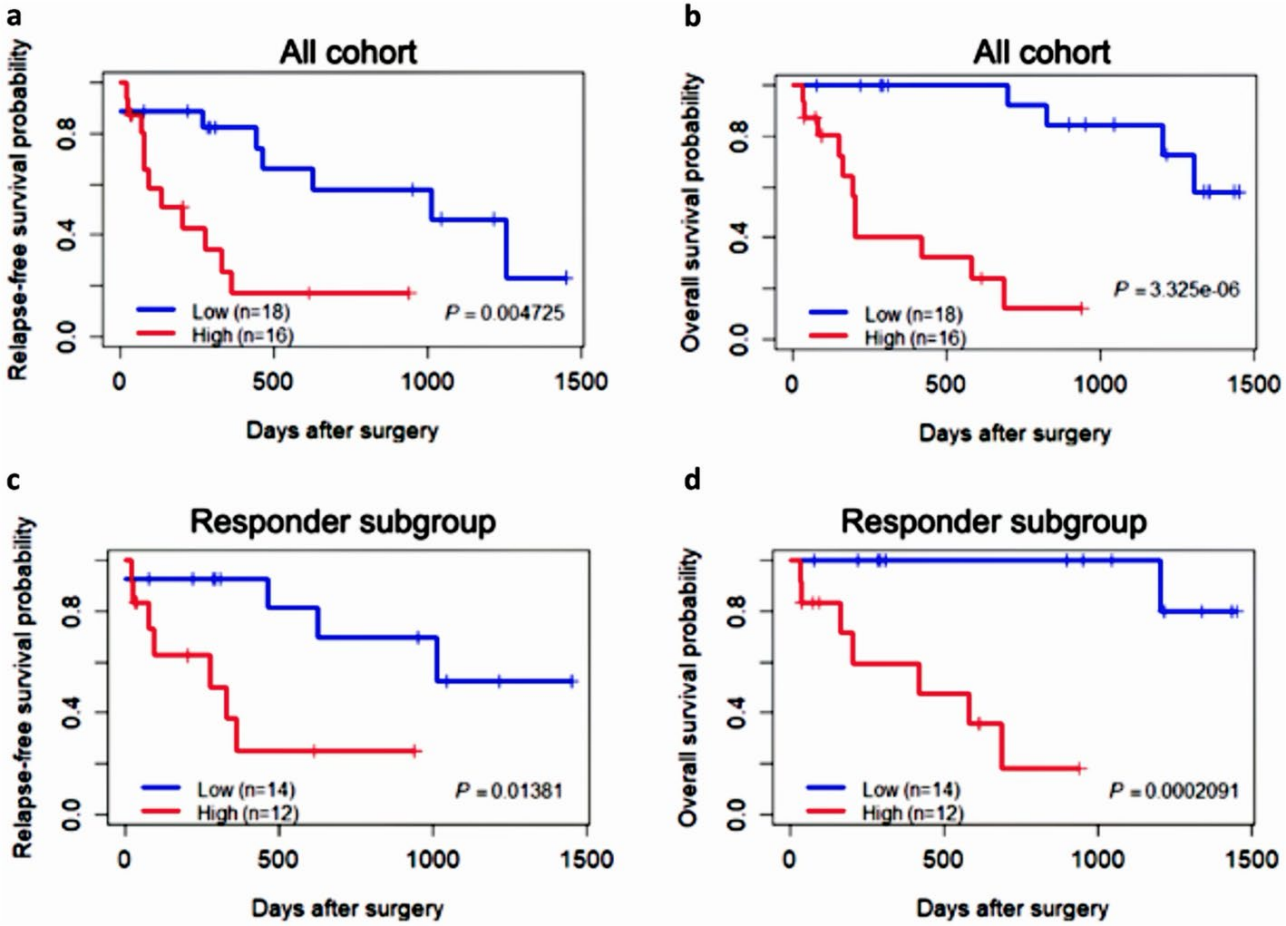
Survival estimates based on mutational gene signature in EAC. Kaplan–Meier plots of low and high-risk score groups based on the median risk score of the cohort (**A**) relapse-free survival and (**B**) overall survival. Kaplan–Meier plots of low and high-risk score groups within patients responded to CRT for (**A**) relapse-free survival and (**B**) overall survival

Using these 9 genes, a mutational gene signature risk score was created with a regression coefficient for overall survival. Using the median mutational risk score as the threshold, it was evident that the 9-gene signature significantly stratified patients into “high-risk” and “low-risk” groups, both for relapse-free survival (*p* = 4.73E-03; Fig. 5A) and overall survival (*p* = 3.325E-06; Fig. 5B). The mutational gene signature also maintained significance in the subgroup of patients who responded to neoadjuvant therapy for relapse-free survival (*p* = 1.38E-02; Fig. 5C) and overall survival (*p* = 2.09E-04; Fig. 5D) as well.

To confirm the prediction accuracy for overall survival, the ROC analysis revealed that mutational gene signature can predict overall survival in treatment naïve biopsies with a higher sensitivity of 73.33% and a specificity of 94.74% (AUC = 0.868, *p* < 0.0001) – Fig. 4B.

## Discussion

The study most importantly presents results from a genetic analysis for patients with locally advanced EAC who received neoadjuvant carboplatin/paclitaxel-based CRT, CROSS regimen. MKI67, SYNE1, PCLO, MSH3, RECQL4, NOTCH2, ILR7, CIITA, LRRK2, and EML4 genes were frequently mutated in patients who respond to CROSS regimen with the tumor mutation count significantly decreased from pre- to post-treatment tumor samples. Similarly, mutations in NLRP1, ALK, and MAP3K1 were frequent among non-responders and tumor mutation count was significantly reduced in post-treatment samples. Previously, pre-treatment high tumor mutational burden (TMB) in EC has been associated with decreased overall survival in patients who did not receive radiation therapy compared to those who did receive radiation therapy, suggesting its use as a predictive marker [23]. Many recent studies have also found a correlation between pre-treatment high TMB and response to immune checkpoint inhibition in solid tumors other than EC. The rationale is that high TMB increases the probability of tumor neoantigen production, which in turn increases the likelihood of immune cell recognition and tumor cell killing [24]. However, in our dataset, we did not observe a correlation between pre-treatment tumor mutation count, for a custom gene panel, and survival outcomes warranting the development of better tools to predict response in EAC patients.

Transitioning to the predictive model, we have determined that mutations in EPHA5, BCL6, and ERBB2 predict response to neoadjuvant therapy in these patients and have created a mutational signature using these genes that more robustly predicts response to CROSS regimen. ERBB2 is synonymous with the Her-2/neu gene and is a well-studied gene. It encodes a receptor tyrosine kinase and amplification of the gene activates the cell-signaling cascade involving the PI3K/AKT/mTOR pathway, which has been associated with the development of breast, gynecologic, and GI malignancies [25]. Before the development of targeted therapies, overexpression of ERBB2 was a negative prognostic marker, but more recently it has been linked to increased pCR rates to neoadjuvant chemotherapy in breast cancer [26, 27]. The clinical role of Trastuzumab as targeted therapy in ERBB2 gene-amplified metastatic gastric or gastro-esophageal junction cancer was established by the ToGA Trial, showing significantly improved overall survival and a 26% reduction in death rate when adding trastuzumab to chemotherapy, but this benefit was not maintained in the neoadjuvant setting [28, 29]. The other two genes included in the model have not yet been shown to have a specific role in the treatment of EC. Alterations in BCL6 are mainly associated with B-cell lymphomas, however, in vitro upregulation of BCL6 has been identified as a possible regulator of response to therapy in EC through inhibition of transcription [30]. While EPHA5 is known to function in cancer in a variety of ways including via the epithelial-to-mesenchymal transition and upregulating cancer stem cell-related markers [31]. It has been shown to mediate trastuzumab resistance in Her-2/neu amplified breast cancers but in other clinical trials has been associated with increased response to different therapies such as immune checkpoint inhibition in lung adenocarcinoma, impacting radiosensitivity in esophageal squamous cell carcinoma (ESCC), and maintaining durable treatment response in metastatic cancer [32–35].

We also found that mutations in APC, MAP3K6, PML, ETS1, CSF3, PDGFRB, GATA2, ARID1A, and FGF6 were associated with overall survival. Using these 9 genes, we have built a prognostic gene signature that can significantly stratify patients into high and low-risk groups based on mutation status for relapse-free and overall survival. The prognostic gene signature holds true in a subgroup of responders as well. Out of the genes described above, only APC, PML, and ARID1A have been linked to survival outcomes in EC. These are all tumor suppressors but are theorized to affect prognosis in different ways. The APC locus shows frequent loss of heterozygosity in EC in at least one study, hypermethylation of the promoter region has been associated with significantly reduced patient survival [36]. APC functions through the WNT signaling pathway, which alters the transcription of target genes such as c-MYC and Cyclin D1 [37]. Previously, loss of ARID1A expression and subsequent mismatch repair insufficiency is associated with improved overall survival in EAC patients, similar to mismatch repair in other malignancies [38]. While PML that directly interacts with p53, acting as a transcriptional co-activator [39], has been established as an independent prognostic classifier for ESCC [40]. The other genes described are not known to prognosticate in EC, although some are known to promote carcinogenesis. For instance, MAP3K6 controls angiogenesis and tumorigenesis under normoxic and hypoxic conditions through VEG-F expression [41], increased levels of PDGFRB have been linked to decreased overall survival in gastric cancer patients, GATA2 defects can cause myelodysplasia and leukemia, and FGF6 defects are associated with the development of prostate and colorectal cancers [42–46].

Given that 5-year overall survival can be as low as 15%, pre-operative CRT has been implemented as a standard to improve progression-free and overall survival in patients with locally advanced EAC. Older studies used cisplatin/5-FU-based chemoradiation with radiation doses ranging from 40–50. 4 Gy, showing improved overall survival and pCR rates of up to 40% [47, 48]. Whereas, patients in the CROSS Trial with node-positive or T2-T3 tumors receiving neoadjuvant carboplatin, paclitaxel and radiation demonstrated a 5-year overall survival rate of 47% and a reduction in locoregional and systemic (to a lesser extent) recurrences [49]. However, failure risk remains significant in this population of patients, particularly distant failures in those who do not achieve pCR [50]. More recently, immunotherapy has been evaluated in the CheckMate 577 study showing that in patients who did not achieve pCR after trimodality therapy, the use of adjuvant Nivolumab significantly improved disease-free survival (22.4 months) compared with observation (11 months) with acceptable toxicity and similar overall health status between the patient groups [51]. This has since been included as an NCCN category 1 recommendation for this subset of patients, but for other patients, there is no standard recommendation regarding adjuvant therapy to decrease the risk of recurrence [4]. Unfortunately, observation till progression remains the most common management approach.

Overall, our findings provide significant potential for personalized management of patients with locally advanced EAC. The predictive panel can help identify patients who may not respond well to neoadjuvant treatment and might benefit from the escalation of therapy using novel chemotherapy and/or immunotherapy combinations to improve response rates and survival outcomes (57). On the other hand, those who are good responders may benefit from de-escalation, and toxicity/quality of life can be improved. Additionally, the prognostic gene signature allows for the stratification of patients into low and high-risk categories. This is especially important for those who have achieved a pCR but are still progressing due to occult metastatic disease. These patients can benefit from adjuvant therapies instead of the most likely recommendation of observation. However, given our small sample size, the predictive and prognostic gene signatures should be prospectively validated in a larger number of patients in a clinical trial setting, before routine use. Additionally, our reported outcomes for trimodality therapy, such as pCR rates and clear resection margin percentages, closely mirrored the CROSS trial results, suggesting that we captured a representative locally advanced EC population (5).

## Conclusions

In conclusion, in patients with locally advanced EAC receiving neoadjuvant chemoradiation with carboplatin/paclitaxel, we have identified a 3-gene signature (EPHA5, BCL6, and ERBB2) that robustly predicts resistance or response to therapy and a 9-gene classifier that significantly prognosticates for improved or worse survival. In the future, these gene panels should be validated in prospective clinical trials as patient risk stratification tools, with the intent to better guide therapeutic decision-making.

## Supplementary Information

**Additional file 1. **

## Data Availability

The analytic tools, approaches and data are all included in the body of the manuscript. Raw datasets used and/or analyzed during the current study are available from the corresponding author on reasonable request.

## Abbreviations

EC: Esophageal cancer
EAC: Esophageal Adenocarcinoma
CRT: Chemoradiotherapy
pCR: Pathologic complete response
R0: Complete resection
DIN: DNA integrity number
PCR: Polymerase chain reaction
GRCh37: Human genome reference hg19
VCF: Variant call format
MAF: Mutation annotation format
OS: Overall survival
ROC: Receiver operating characteristic curve
AUC: Area under the curve
TMB: Tumor mutational burden
ESCC: Esophageal squamous cell carcinoma
