# Supplementary material for: Prognostic and predictive biomarkers for response to neoadjuvant chemoradiation in esophageal adenocarcinoma
Source: Biomark Res. 2022 Nov 14;10:81. doi: 10.1186/s40364-022-00429-6 (PMC9664643; doi:10.1186/s40364-022-00429-6)
Supplement: Supplementary file 1 — Additional file 1. [file 40364_2022_429_MOESM1_ESM.pdf]

APPENDIX A 640 COMPREHENSIVE GENE PANEL

ABL1	BCOR	CDK8	DTX1	FGF10	HDAC4	ITGA9	MAPK1	NCOA4	PDGFRB	RB1	SOX11	TP63
ABL2	BCORL1	CDKN1B	DUSP2	FGF14	HDAC7	ITGB2	MAPK8	NCOR1	PDK1	RBM15	SOX2	TPR
ACTB	BCR	CDKN2A	DUSP9	FGF19	HGF	ITGB3	MARK1	NCOR2	PER1	RECQL4	SOX9	TRAF2
ACVR1B	BIRC2	CDKN2B	EBF1	FGF23	HIF1A	JAK1	MARK4	NCSTN	PGAP3	REL	SPEN	TRAF3
ACVR2A	BIRC3	CDKN2C	ECT2L	FGF3	HIST1H1C	JAK2	MBD1	NF1	PHF6	RELN	SPI1	TRAF5
ADAMTS20	BIRC5	CEBPA	EED	FGF4	HIST1H1D	JAK3	MCL1	NF2	PHOX2B	RET	SPOP	TRAF7
AFF1	BLM	CEBPB	EGFR	FGF6	HIST1H1E	JARID2	MDM2	NFE2L2	PICALM	RHOA	SRC	TRIM24
AFF3	BLNK	CHD2	ELP2	FGFR1	HIST1H2AC	JUN	MDM4	NFKB1	PIGA	RHOH	SRSF2	TRIM33
AKAP9	BMPR1A	CHEK1	EML4	FGFR2	HIST1H2AG	KAT6A	MED12	NFKB2	PIK3C2B	RICTOR	SSX1	TRIP11
AKT1	BRAF	CHEK2	EP300	FGFR3	HIST1H2AL	KAT6B	MEF2B	NFKBIA	PIK3CA	RIT1	STAG2	TRRAP
AKT2	BRCA1	CHIC2	EP400	FGFR4	HIST1H2AM	KDM2B	MEF2C	NIN	PIK3CB	RNASEL	STAT3	TSC1
AKT3	BRCA2	CIC	EPHA3	FH	HIST1H2BC	KDM4C	MEN1	NKX2-1	PIK3CD	RNF2	STAT4	TSC2
ALK	BRD3	CIITA	EPHA5	FHIT	HIST1H2BJ	KDM5A	MET	NLRP1	PIK3CG	RNF213	STAT5A	TSHR
APC	BRD4	CKS1B	EPHA7	FLCN	HIST1H2BK	KDM5C	MIB1	NOD1	PIK3R1	RNF43	STAT5B	TUSC3
APH1A	BRIP1	CMPK1	EPHB1	FLI1	HIST1H2BO	KDM6A	MITF	NOTCH1	PIK3R2	ROS1	STAT6	TYK2
AR	BRSK1	COL1A1	EPHB4	FLT1	HIST1H3B	KDR	MKI67	NOTCH2	PIM1	RPN1	STK11	U2AF1
ARAF	BTG2	CPS1	EPHB6	FLT3	HLF	KEAP1	MKL1	NOTCH4	PKHD1	RPS6KA2	STK36	U2AF2
ARFRP1	BTK	CRBN	ERBB2	FLT4	HNF1A	KIT	MLH1	NPM1	PLAG1	RPTOR	SUFU	UBR5
ARHGAP26	BTLA	CREB1	ERBB3	FLYWCH1	HOOK3	KLF4	MLL	NRAS	PLCG1	RRM1	SUZ12	UGT1A1
ARID1A	BUB1B	CREBBP	ERBB4	FN1	HOXA11	KLF6	MLL2(KMT2D)	NSD1	PLCG2	RUNX1	SYK	USP9X
ARID1B	C11ORF30	CRKL	ERCC1	FOXL2	HOXA13	KLHL6	MLL3	NT5C2	PLEKHG5	RUNX1T1	SYNE1	VHL
ARID2	CAD	CRLF2	ERCC2	FOXO1	HOXA9	KMT2A	MLLT10	NTRK1	PML	S1PR2	TAF1	WAS
ARNT	CALR	CRLF2	ERCC3	FOXO3	HRAS	KMT2B	MLLT4	NTRK2	PMS1	SAMD9	TAF1L	WDR90
ASMTL	CARD11	CRTC1	ERCC4	FOXP1	HSP90AA1	KMT2C	MLLT6	NTRK3	PMS2	SBDS	TAL1	WHSC1
ASMTL	CASC5	CSF1R	ERCC5	FOXP4	HSP90AB1	KRAS	MMP2	NUMA1	POT1	SDHA	TBL1XR1	WISP3
ASXL1	CASP8	CSF3R	ERG	FRS2	ICK	LAMP1	MN1	NUP214	POU5F1	SDHB	TBX22	WRN
ATF1	CBFB	CSMD3	ESR1	FUBP1	ID3	LCK	MNX1	NUP93	PPARG	SDHC	TCF12	WT1
ATM	CBL	CTCF	ETNK1	FZR1	IDH1	LEF1	MPL	NUP98	PPP2R1A	SDHD	TCF3	XBP1
ATR	CBLB	CTNNA1	ETS1	G6PD	IDH2	LIFR	MRE11A	P2RY8	PRDM1	SEPT9	TCF7L1	XPA
ATRX	CCND1	CTNNB1	ETV1	GADD45B	IGF1R	LMO1	MSH2	P2RY8	PRDM16	SERP2	TCF7L2	XPC
AURKA	CCND2	CUX1	ETV4	GATA1	IGF2	LPHN3	MSH3	PAG1	PRKAR1A	SETBP1	TCL1A	XPO1
AURKB	CCND3	CXCR4	ETV6	GATA2	IGF2R	LPP	MSH6	PAK3	PRKDC	SETD2	TET1	XRCC2
AURKC	CCNE1	CYLD	EXOSC6	GATA3	IKBKB	LRP1B	MTOR	PALB2	PRSS8	SF3B1	TET2	YY1AP1
AXIN1	CCT6B	CYP2C19	EXT1	GDNF	IKBKE	LRRK2	MTR	PARP1	PSIP1	SGK1	TFE3	ZBTB16
AXL	CD22	CYP2D6	EXT2	GID4	IKZF1	LTF	MTRR	PASK	PTCH1	SH2D1A	TGFBR2	ZMYM3
B2M	CD274	DAXX	EZH2	GNA11	IKZF2	LTK	MUC1	PAX3	PTEN	SH2B3	TGM7	ZNF217
BAI3	CD36	DCC	FAF1	GNA12	IKZF3	MAF	MUTYH	PAX5	PTGS2	SKP2	THBS1	ZNF24
BANF1	CD58	DDB2	FAM123B	GNA13	IL2	MAFB	MYB	PAX7	PTPN11	SMAD2	TIMP3	ZNF384
BAP1	CD70	DDIT3	FAM46C	GNAQ	IL21R	MAGEA1	MYC	PAX8	PTPN2	SMAD4	TLL2	ZNF521
BARD1	CD79A	DDR2	FANCA	GNAS	IL6ST	MAGED1	MYCL1	PBRM1	PTPN6	SMARCA1	TLR4	ZNF703
BCL10	CD79B	DDX10	FANCC	GPR124	IL7R	MAGI1	MYCN	PBX1	PTPRD	SMARCA4	TLX1	ZRSR2
BCL11A	CDC73	DDX3X	FANCD2	GRIN2A	ING4	MALT1	MYD88	PC	PTPRO	SMARCB1	TMEM30A	
BCL11B	CDH1	DEK	FANCE	GRM8	INHBA	MAML2	MYH11	PCBP1	PTPRT	SMC1A	TMSL3	
BCL2	CDH11	DICER1	FANCF	GSK3B	INPP4B	MAP2K1	MYH9	PCLO	RAD21	SMC3	TNFAIP3	
BCL2L1	CDH2	DNM2	FANCG	GSX2	INPP5D	MAP2K2	MYO18A	PDCD1	RAD50	SMO	TNFRSF11A	
BCL2L2	CDH20	DNMT1	FANCL	GTSE1	IRF1	MAP2K4	MYST3	PDCD11	RAD51	SMUG1	TNFRSF14	
BCL3	CDH5	DNMT3A	FAS	GUCY1A2	IRF4	MAP3K1	NBN	PDCD1LG2	RAF1	SOCS1	TNFRSF17	
BCL6	CDK12	DOT1L	FBXO11	H3F3A	IRF8	MAP3K14	NCOA1	PDE4DIP	RALGDS	SOCS2	TNK2	
BCL7A	CDK4	DPYD	FBXO31	HCAR1	IRS2	MAP3K6	NCOA2	PDGFB	RARA	SOCS3	TOP1	
BCL9	CDK6	DST	FBXW7	HDAC1	ITGA10	MAP3K7	NCOA3	PDGFRA	RASGEF1A	SOX10	TP53	

Along with Intronic region(s) which include the following SNP's of interest: TERTre2736100, TERTrs2853677 and ATMrs1800056
